# Potential Impact of Global Warming on Virus Propagation in Infected Plants and Agricultural Productivity

**DOI:** 10.3389/fpls.2021.649768

**Published:** 2021-03-31

**Authors:** Khalid Amari, Caiping Huang, Manfred Heinlein

**Affiliations:** Institute of Plant Molecular Biology (IBMP), CNRS UPR 2357, Université de Strasbourg, Strasbourg, France

**Keywords:** plant viruses, *Tobacco mosaic virus*, temperature, global warming, agriculture, plasmodesmata, tolerance

## Abstract

The increasing pace of global warming and climate instability will challenge the management of pests and diseases of cultivated plants. Several reports have shown that increases in environmental temperature can enhance the cell-to-cell and systemic propagation of viruses within their infected hosts. These observations suggest that earlier and longer periods of warmer weather may cause important changes in the interaction between viruses and their host’s plants, thus posing risks of new viral diseases and outbreaks in agriculture and the wild. As viruses target plasmodesmata (PD) for cell-to-cell spread, these cell wall pores may play yet unknown roles in the temperature-sensitive regulation of intercellular communication and virus infection. Understanding the temperature-sensitive mechanisms in plant-virus interactions will provide important knowledge for protecting crops against diseases in a warmer climate.

## Introduction

**FIGURE 1:**
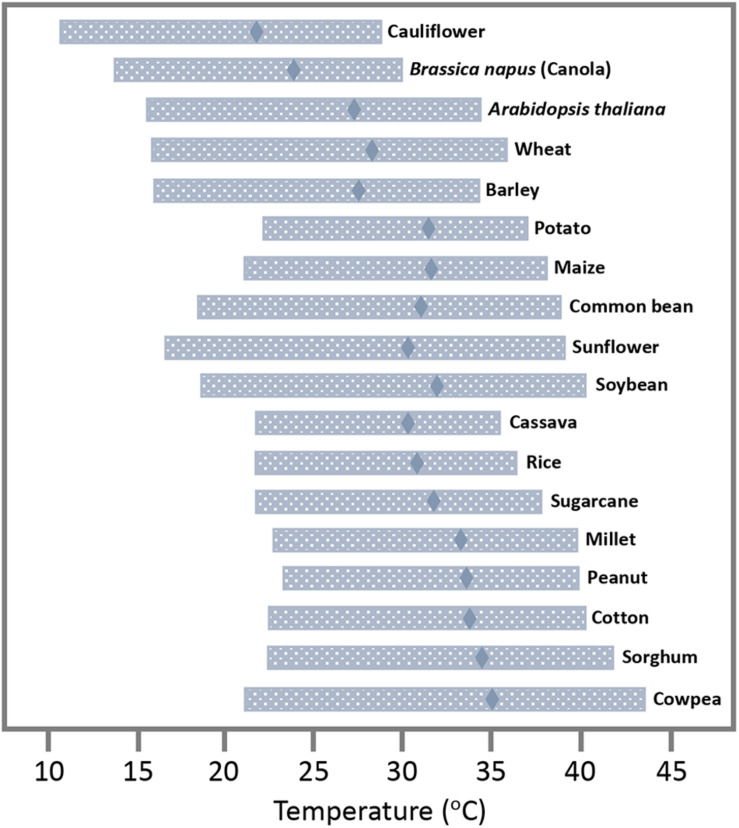
Temperature tolerance range of plant and crop species. Temperature optimum at which plant development has its maximum rate (diamonds) and range of temperature for which the rate of development is at least 50% of its maximum (horizontal bars). Adapted from Parent and Tardieu (2012), with permission by the authors.

Viruses can cause major losses in crop yields and are the primary cause of emerging diseases in plants (Nicaise, 2014). As has been reviewed comprehensively, changes in temperature and other parameters of climate change (changes in rainfall patterns, wind, accumulation of greenhouse gases, and extreme weather events, to name a few) are expected to affect the geographic distribution of the viral hosts and vectors, and thus the epidemiology of viruses that depend on these hosts and vectors for propagation and inter-plant transmission (Canto et al., 2009; Jones and Barbetti, 2012). As global temperatures increase, poleward and higher altitude areas with currently colder weather likely assume a more temperate climate, whereas the regions with currently temperate climate become warmer and may assume a climate that is more typical for tropical zones. Thus, in response to global temperature increases, the viral hosts and vectors adapted to temperate climates are expected to spread with their viruses poleward and to higher altitudes where temperatures will then be temperate, whereas hosts and viral vectors in tropical regions will invade with their viruses the regions with a currently mild climate where temperatures may have increased toward values that are currently typical for tropical areas. In these newly invaded regions, virus hosts and vectors as well as their viruses may find conditions that are similar as in previous habitats, thus allowing them to interact as previously. From this point of view, a warmer climate is predicted to cause a global shift in the distribution of viruses along with their hosts and vectors. And, because this process may allow viruses to interact with their current hosts and vectors as previously, a strong global impact on virus propagation and virus-caused diseases in agriculture may not be expected. On the other hand, this view may be too simple. First, the new geographical locations may allow viruses to find new hosts, which poses risks of new emerging diseases (Jones, 2020). Second and more important, plants tolerate broad ranges of temperatures (Parent and Tardieu, 2012; Figure 1), and most of them will likely not migrate, at least not immediately. For canola and potato, for example, the ranges of temperatures for growth with at least 50% of the maximum rate are from 15.0 to 29.6°C and from 21.6 to 37.3°C, respectively. For sunflower, which has a temperature optimum around 29°C, the range is from 17.3 to 38.3°C. The average high temperature currently reached in summer in London, Paris, or Berlin (23–25°C;^1^) is below the temperature optima for crops grown in the area such as wheat, barley, potato, maize, or sunflower (27.7, 26.6, 30.6, 30.8, and 29.3°C, respectively; Parent and Tardieu, 2012). Thus, there is room for temperature increases until these and other plants are forced to migrate. According to current climate change projections by the Intergovernmental Panel on Climate Change (IPCC), the global mean surface temperature change for the period 2016–2035 relative to 1986–2005 will likely be in the range of 0.3–0.7°C and, dependent on prediction scenarios, may reach 0.3–4.8°C at the end of the 21st century (2081–2100) (IPCC, 2014). These global changes in temperature are predicted to be accompanied by local weather extremes with heat waves and drought that cause significant yield losses (Bita and Gerats, 2013). However, except for plants currently growing in areas with conditions at their tolerance margins, these predicted changes in temperature will not cause an immediate global migration of all viruses and their hosts away from their current geographical zones. For most hosts and their viruses, expected movements to colder areas may occur only gradually, over long ranges of time. However, while these plants remain at their locations, warmer local temperatures will nevertheless already have immediate effects on the intracellular environment provided by these hosts to viruses, thus posing risks to agriculture.

**FIGURE 2:**
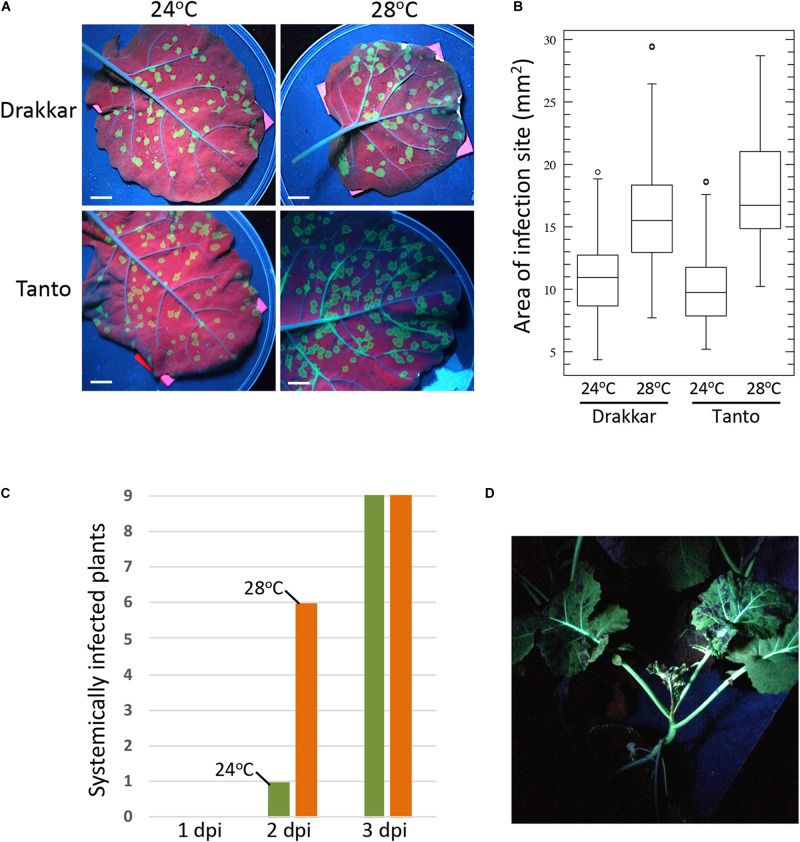
Temperature effect on TuMV-GFP infection in *B. napus* cultivars Drakkar and Tanto. Plants were incubated at 20°C for 8 h (night) and 24°C for 16 h (day) until two leaf stage. Half of the plants were then transferred to 20°C for 8 h (night) and 28°C for 16 h (day). Plants were allowed to adjust for 2 days before inoculation. **(A)** Effect of temperature on viral cell-to-cell spread in inoculated leaves. Pictures were taken at 6 days post inoculation (6 dpi) under UV light. Scale bar, 1 cm. **(B)** Sizes of individual local infection sites at 6 dpi. Infection foci in leaves of five plants per condition were measured (Drakkar 24°C, *N* = 117; Drakkar 28°C, *N* = 111; Tanto 24°C, *N* = 112; Tanto 28°C, *N* = 102. The higher temperature causes a significant increase in the local cell-to-cell spread of infection in both Drakkar (ANOVA, *p* = 4,3^− 21^) and Tanto (ANOVA, *p* = 2,5^− 37^). **(C)** Systemic spread of TuMV-GFP in Drakkar is more efficient at 28°C (orange) than at 24°C (green). Inoculated leaves of nine plants at 24°C and of nine plants at 28°C were removed after 1, 2, or 3 dpi, followed by scoring the systemic leaves for GFP fluorescence (systemic infection) at 16 dpi. A control plant from which the inoculated leaf was not removed is shown in panel **(D)**. **(D)** TuMV-GFP-infected Drakkar plant showing systemic infection at 21 dpi. The picture was taken under UV light.

Temperature is a physical parameter that influences biochemical reactions and higher molecular structures, like DNA and proteins, and supramolecular components, like membranes and the elements of the cytoskeleton, through simple thermodynamic effects (Ruelland and Zachowski, 2010). Warmer temperature thereby causes increased membrane fluidity and cytoskeletal dynamics, which can enhance the propagation of plant viruses that generally depend on these cellular components for replication and spread within their hosts. Consistently, numerous studies demonstrated that the rate at which viruses replicate and move through the infected plant increases with temperature up to a certain temperature optimum, beyond which viral propagation decreases. For example, Lebeurier and Hirth (1966) demonstrated already more than 50 years ago in *Nicotiana tabacum* leaf disks that *Tobacco mosaic virus* (TMV) increases its multiplication with temperature and that replication is again lower only when temperature reaches 34–36°C. Later, with a green fluorescent protein (GFP)-tagged virus it was shown that the cell-to-cell spread in *N. benthamiana* plants was threefold stronger when the temperature was increased by 10 degrees (from 22 to 32°C) (Boyko et al.,2000a,b). Similarly, *Turnip mosaic virus* (TuMV) showed increased accumulation in Chinese cabbage when temperature was increased from 13 to 23–28°C (Chung et al., 2015) and a GFP-tagged version of this virus shows a twofold more efficient cell-to-cell (Figures 2A,B) and systemic (Figures 2C,D) spread in canola (*Brassica napus*) upon shifting the daytime temperature by only four degrees from 24 to 28°C. In Arabidopsis, TuMV was shown to accumulate to higher levels when kept at 25°C during the day and 15°C in the night as compared to colder temperatures with 15°C during the day and 5°C in the night (Honjo et al., 2020). Potato plants infected with *Potato virus Y* showed a dramatic increase in systemic infection when temperatures were increased from 23 to 28°C (Choi et al., 2017). *Barley yellow dwarf virus* (BYDV) showed strong increases in systemic movement in oat when the temperature was elevated from 15.5 to 21°C (Jensen, 1973) and infection of bean leaves with *Rothamsted tobacco necrosis virus* (RTNV) increased with rising temperature from 10 to 22°C (Harrison, 1956). The spread and replication of *Wheat streak mosaic virus* and disease development in Winter Wheat was shown to increase with temperature within the tested temperature range of 10–27°C (Wosula et al., 2017). Given that virus accumulation and disease symptoms are often correlated, these examples hint toward the imminent risk that earlier and longer periods of warmer weather will aggravate virus-induced diseases in crops in their current growing areas, thus endangering yields.

The risks imposed by global warming may not be limited to viruses in crops, however. It is often overlooked that viruses are ubiquitous in the wild and play an important role in the evolution of life. Surveys have shown that 60–70% of plants grown in natural ecosystems are infected with viruses (Roossinck, 2015). Continuous host-virus co-evolution in natural habitats allows viruses to adapt to their hosts and maintain or even improve their fitness. Thus, plants in the wild are normally free of symptoms despite infection by viruses. Although a significant virus load is sustained in such virus-tolerant plants, the plant growth, yield, or reproduction attributes are only minimally affected and visible symptoms are either absent or mild (Pagan and Garcia-Arenal, 2018; Paudel and Sanfacon, 2018). However, tolerance, thus the ability of the infected plant to reduce negative effects of the infection, is a complex and highly evolved trait that depends on a well-adjusted host-virus crosstalk (Kørner et al., 2018; Paudel and Sanfacon, 2018; Pitzalis et al., 2020) and contributes to host fitness through multiple molecular mechanisms (Pagan and Garcia-Arenal, 2018, 2020). Higher average temperatures, leading to a more favorable environment for virus replication and movement, may break the delicate equilibrium between plant viruses and their hosts and thereby lead to the outbreak of new diseases. Such temperature-related loss of tolerance in crops and wild species and the spreading of new diseases from natural reservoirs toward crops may have important consequences for agriculture.

As humanity faces increasing average annual temperatures, it is important to understand the temperature-sensitive mechanisms that determine the propagation and spread of viruses within their hosts. The underlying mechanisms can be of a diverse nature. Apart from increased membrane fluidity and cytoskeletal dynamics that likely accelerate the membrane- and cytoskeleton-associated processes involved in virus replication and transport, temperature may also affect the regulation of the intercellular communication channels in the plant cell walls (plasmodesmata). Plasmodesmata (PD) are important gates through which viruses must move their genomes to spread infection between cells, into the phloem, and finally throughout the plant. In accordance with their central function in intercellular communication, PD are equipped with receptor proteins and receptor protein kinases that form signaling hubs through which PD are enabled to orchestrate processes related to plant growth and development but also responses to pathogens and abiotic stresses (Lee, 2015; Stahl and Faulkner, 2016). A recent study highlights the ability of PD to recruit receptor-like kinases in response to osmotic stress (Grison et al., 2019). It seems feasible, therefore, that changes in temperature can lead to specific alterations in the composition and regulation of PD, which in turn likely affects virus spread from cell to cell. So far, there are only few studies addressing the specific effects of temperature on PD function. Studies in poplar revealed that temperature influences the expression of dormancy-related genes, including gibberellin-acid (GA)-inducible members of beta-1,3-glucanase family involved in the degradation of callose at the plasmodesmal dormancy sphincter complexes (Rinne et al., 2011, 2018). Effects of temperature on PD structure and conductivity were also observed in maize (Bilska and Sowinski, 2010). Recent progress made in the analysis of PD structure, composition, and regulation (Wu et al., 2018; Brault et al., 2019; Grison et al., 2019; Huang et al., 2019; Cheval et al., 2020; Iswanto et al., 2020; Liu et al., 2020) may facilitate future research in model systems such as *N. benthamiana* and Arabidopsis to reveal the impact of increasing temperature on PD proteins and membranes and whether such changes correlate with an altered flow of PD targeted and non-targeted macromolecules (e.g., GFP) through the pore.

Warmer temperature may facilitate virus spread also by altering the activity or turnover of the viral movement proteins (MPs), which may affect the interaction of viruses with PD. The mentioned increased efficiency of intercellular spread of TMV in *N. benthamiana* at higher temperature correlated with changes in the subcellular accumulation pattern of the virus-encoded, GFP-tagged MP (Boyko et al., 2000b). During infection the protein is expressed in distinct cortical endoplasmic reticulum (cortical ER)-associated replication complexes that are formed at sites of the cortical ER at which this membrane network intersects with cortical microtubules (Niehl et al., 2013). At the lower temperature (22°C) the MP tends to stay and over-accumulate in the replication complexes that can grow to large sizes over time (Padgett et al., 1996; Heinlein et al., 1998; Niehl et al., 2013; Heinlein, 2015). At the higher temperature (32°C), however, the MP accumulates along microtubules rather than in the replication complexes (Boyko et al., 2000b). The ability of MP to interact with microtubules is involved in virus movement and has been correlated with the formation and mobility of distinct MP-containing replication complexes during early stages of infection in cells at the infection front (Boyko et al., 2000a, 2007; Niehl et al., 2014). Accumulation of high amounts of MP along the length of microtubules, in contrast, is dispensable for movement and is rather linked to its degradation in cells having completed the movement process (Gillespie et al., 2002; Niehl et al., 2012). The degradation of MP is triggered by its accumulation in the ER and depends on CDC48, an ATP-driven machinery that controls ER homeostasis by extracting over-accumulating or misfolded proteins from the membrane (Niehl et al., 2012). These observations are important in the context of other findings indicating that ER-associated virus replication and viral protein accumulation cause ER stress (Park and Park, 2019) and can lead to PD closure (Guenoune-Gelbart et al., 2008), thereby causing resistance against virus movement. Thus, by enhancing the removal of over-accumulated MP from the ER, which thereby results in the accumulated binding of MP along microtubules and promotes MP degradation, warmer temperature may avoid ER stress and PD closure, thereby facilitating efficient virus movement. Because of its direct binding affinity for microtubules (Ashby et al., 2006), the extraction of accumulated MP from the ER leads to its alignment along microtubules, which is directly or indirectly supported by another microtubule-associated protein (Curin et al., 2007). It is noteworthy that the movement of the TMV-related Oilseed rape mosaic virus (ORMV, also known as Youcai mosaic virus or TMV-Cg) is associated with the formation of distinct MP-containing, replication complexes during early stages of infection just like in the case of TMV. However, unlike the MP of TMV, the MP of ORMV does not accumulate on the ER or along microtubules. Importantly, this MP allows faster virus movement than the MP of TMV (Niehl et al., 2014). These observations are consistent with the conclusion that the enhanced TMV movement at higher temperature is associated with specific MP activities and turnover conditions.

Warmer temperature could affect TMV movement also by increasing myosin motor activity, thus facilitating the myosin-driven transport of the virus and of MP along the ER-actin network to the PD (Hofmann et al., 2009; Amari et al., 2014). Temperature may also alter the ability of MP to bind RNA and other proteins, or to gate the PD channel. Potentially, such alterations in MP activity could be mediated through changes in the post-translational phosphorylation of the protein (Trutnyeva et al., 2005).

However, warmer temperatures may affect virus cell-to-cell movement also more indirectly, for example, by causing changes in gene expression and alterations in the interactions of viruses with host defense responses. In plants carrying specific resistance (R) genes, warmer temperature may indeed provoke stronger infections since resistance genes against biotrophic and hemi-biotrophic microbes (viruses, bacteria, fungi) are often temperature-sensitive and are inactivated at elevated temperature (Wang et al., 2009). Thus, in several plant-virus interactions, hypersensitive resistance (HR) or HR-like responses are slower when the temperature is elevated by a few degrees from 21–22°C to 27–28°C, and are lost at temperatures above 30°C (Whitham et al., 1996; Wang et al., 2009). In addition to R-gene mediated resistance, plants control their viruses also through pattern-triggered immunity (PTI) (Kørner et al., 2013; Niehl et al., 2016; Teixeira et al., 2019; Amari and Niehl, 2020) and RNA silencing (Ding, 2010; Pumplin and Voinnet, 2013). Both defense pathways are activated by dsRNA produced during virus infection. PTI involves the activation of transcriptional signaling that confers broad-spectrum pathogen resistance. In contrast, RNA silencing uses 21–24 nts long small interfering RNAs (siRNAs) and 21 nts microRNAs (miRNAs) to direct sequence-specific cleavage or translational repression of viral and host RNAs through ARGONAUTE (AGO)-containing RNA silencing effector complexes. While antiviral PTI has only recently been discovered and its sensitivity to temperature in the context of virus infection remains to be studied, RNA silencing has been reported to be more active at higher temperature and shown to be correlated with reduced disease symptoms in infected tissues (Szittya et al., 2003). However, other reports argue against such correlations or even conclude that RNA silencing or its systemic signaling is inhibited at elevated temperature (Zhong et al., 2013). These contrasting findings show that we are far from understanding how temperature influences the interaction of viruses with host defense pathways and that further studies are needed.

As already highlighted, global warming comes along with various other impacts on humidity, drought, rainfall intensity and rainfall patterns, wind speed and direction, and greenhouse gas concentration. These parameters will result in altered crop cultivations systems and the range of cultivated species grown. This, in turn, will also influence the distribution of the viral insect vectors and plant hosts and, thereby, the distribution and evolution of virus species. However, while these long-term global consequences of climate change need to be studied, monitored, and modeled to improve disease management, changes in temperature have immediate effects on cellular mechanisms that are at the core of plant-virus interactions within each infected cell, irrespective of the region where the infected host is grown. This may aggravate diseases in natural and agricultural settings and damage yields and crop survival before temperatures will eventually exceed the host temperature tolerance ranges. To ensure agricultural yield, a concerted research effort is needed to understand the cellular mechanisms that determine disease tolerance in infected plants and to use this knowledge to adapt our crops toward temperature resilience and tolerance for viruses through dedicated breeding programs.

## Data Availability Statement

The original contributions presented in the study are included in the article/supplementary material, further inquiries can be directed to the corresponding author/s.

## Author Contributions

CH and MH wrote the manuscript. For Figure 2, MH conceived and designed the work. KA performed the acquisition and analysis of the data. All authors edited and approved the final version of the manuscript and agreed to be accountable for all aspects of the work.

## Conflict of Interest

The authors declare that the research was conducted in the absence of any commercial or financial relationships that could be construed as a potential conflict of interest.

## Footnotes

1 https://www.weather-atlas.com/en/
